# Restoring the quantity and quality of elderly human mesenchymal stem cells for autologous cell-based therapies

**DOI:** 10.1186/s13287-017-0688-x

**Published:** 2017-10-27

**Authors:** Travis J. Block, Milos Marinkovic, Olivia N. Tran, Aaron O. Gonzalez, Amanda Marshall, David D. Dean, Xiao-Dong Chen

**Affiliations:** 1 Department of Comprehensive Dentistry, University of Texas Health Science Center at San Antonio, 7703 Floyd Curl Drive, San Antonio, TX 78229-3900 USA; 2 Department of Biomedical Engineering, University of Texas at San Antonio, San Antonio, TX 78249 USA; 3 San Antonio Orthopaedic Specialists, San Antonio, TX 78258 USA; 4 Audie Murphy VA Medical Center, San Antonio, TX 78229 USA

**Keywords:** Aging, Stem cell markers, Cellular senescence, Senescence-associated secretory phenotype, Extracellular matrix, Stem cell niche

## Abstract

**Background:**

Degenerative diseases are a major public health concern for the aging population and mesenchymal stem cells (MSCs) have great potential for treating many of these diseases. However, the quantity and quality of MSCs declines with aging, limiting the potential efficacy of autologous MSCs for treating the elderly population.

**Methods:**

Human bone marrow (BM)-derived MSCs from young and elderly donors were obtained and characterized using standard cell surface marker criteria (CD73, CD90, CD105) as recommended by the International Society for Cellular Therapy (ISCT). The elderly MSC population was isolated into four subpopulations based on size and stage-specific embryonic antigen-4 (SSEA-4) expression using fluorescence-activated cell sorting (FACS), and subpopulations were compared to the unfractionated young and elderly MSCs using assays that evaluate MSC proliferation, quality, morphology, intracellular reactive oxygen species, β-galactosidase expression, and adenosine triphosphate (ATP) content.

**Results:**

The ISCT-recommended cell surface markers failed to detect any differences between young and elderly MSCs. Here, we report that elderly MSCs were larger in size and displayed substantially higher concentrations of intracellular reactive oxygen species and β-galactosidase expression and lower amounts of ATP and SSEA-4 expression. Based on these findings, cell size and SSEA-4 expression were used to separate the elderly MSCs into four subpopulations by FACS. The original populations (young and elderly MSCs), as well as the four subpopulations, were then characterized before and after culture on tissue culture plastic and BM-derived extracellular matrix (BM-ECM). The small SSEA-4-positive subpopulation representing ~ 8% of the original elderly MSC population exhibited a “youthful” phenotype that was similar to that of young MSCs. The biological activity of this elderly subpopulation was inhibited by senescence-associated factors produced by the unfractionated parent population. After these “youthful” cells were isolated and expanded (three passages) on a “young microenvironment” (i.e., BM-ECM produced by BM cells from young donors), the number of cells increased ≈ 17,000-fold to 3 × 10^9^ cells and retained their “youthful” phenotype.

**Conclusions:**

These results suggest that it is feasible to obtain large numbers of high-quality autologous MSCs from the elderly population and establish personal stem cell banks that will allow serial infusions of “rejuvenated” MSCs for treating age-related diseases.

**Electronic supplementary material:**

The online version of this article (doi:10.1186/s13287-017-0688-x) contains supplementary material, which is available to authorized users.

## Background

Because of increased life expectancy, age-related degenerative diseases are becoming an important public health concern [1, 2]. This increase in the frequency of degenerative disease has coincided with the advent of regenerative medicine-based tools for creating stem cell-based therapies. Although researchers have actively pursued stem cell-based therapies for retarding or reversing age-related degeneration [3, 4], clinical trials aimed at demonstrating stem cell efficacy have produced inconsistent results [5, 6].

The microenvironment (or niche), where stem cells normally reside, is known to have a major impact on stem cell function [7]. In the laboratory, stem cell behavior is often evaluated in tissue culture plastic (TCP) vessels, where extrinsic factors typically present in the niche are missing. Clearly, our view of how stem cell behavior is regulated must include the combined effects of both extrinsic factors (e.g., growth factors, extracellular matrix (ECM), and immune cells) and various intrinsic properties of the stem cells themselves [8–10]. These considerations are especially important when developing stem cell-based therapies for age-related degenerative diseases because the cells must be able to function in a predictable manner while residing in a microenvironment damaged by aging or disease [11, 12].

Our laboratory was the first to describe the production of a native three-dimensional (3D) decellularized bone marrow-derived extracellular matrix (BM-ECM) culture system which mimics the stem cell microenvironment in vivo and provides many of the critical biochemical and physical cues for initiating and sustaining cell functions [8]. Mouse and human BM-MSCs, cultured on these ECMs, display enhanced attachment and proliferation, while retaining their stem cell properties [13, 14]. In more recent work, we demonstrated that culture on BM-ECM, produced by young mouse stromal cells, restores youthful replication and osteogenic potential of MSCs obtained from elderly mice [15]. The many advantages of maintaining MSCs on a native 3D ECM have been recognized by a number of other groups [16].

Autologous stem cell-based therapies are preferable due to biosafety concerns. In addition, increasing evidence suggests that MSCs may not be immune privileged [17, 18]. Unfortunately, autologous MSC-based therapies have been impeded by the fact that MSC quantity and quality decline with aging [19]. Since elderly patients are the primary target population for cell-based treatment of age-related diseases, it is essential that a reproducible strategy for providing sufficient quantities of high-quality autologous cells is developed and rigorously tested.

Prior studies have demonstrated that the clonal composition of hematopoietic stem cell (HSC) populations, rather than individual stem cells, change with aging [20]. If this was also found to be true for MSCs, it would suggest the possibility of being able to harvest “youthful” cells from elderly donors. Several lines of evidence support this idea, including reports demonstrating that elderly MSC populations consist of mixtures of cells and contain senescent cells which produce factors that inhibit healthy cells [21–23].

In the present study, we propose that the relative ratio of “youthful” to elderly (“aged”) cells in the BM-MSC population reverses with aging and that old MSCs not only lose their self-renewal and differentiation capacity, but also release harmful factors that suppress the youthful subpopulation of elderly MSCs. These changes result in an inexorable functional decline of the overall elderly MSC population. For the current study, cells from 11 randomly selected male donors, out of a repository of elderly MSCs collected from 119 donors (aged 60–96 years), were used to compare/contrast the characteristics of young and elderly MSCs and determine whether it is possible to rejuvenate elderly MSCs. Here, we test the hypothesis that high-quality MSCs can be rescued from elderly populations by first isolating a subpopulation of “youthful” cells and then expanding these cells on a “young microenvironment”. The results are provocative and suggest that it may be feasible to bank large quantities of high-quality autologous MSCs from the elderly population for treatment of age-related diseases.

## Methods

### BM from young donors

BM from five healthy, male donors (age < 23 years) was obtained with informed consent from LONZA (Walkersville, MD, USA; see company website for IRB information). Fresh, unprocessed samples were received from the supplier, seeded into TCP vessels (5 × 10^5^ cells/cm^2^), and cultured in “growth media” as described previously [8, 10]. Cells were expanded for one or two passages (P1/P2) and then used in the experiments or stored in liquid nitrogen.

### BM from elderly donors

BM cells from elderly donors (age 65 years or older) were obtained with UTHSCSA IRB approval from consenting patients undergoing total knee or hip arthroplasty. Routinely discarded cancellous bone from the surgical site was removed and immediately placed in isolation buffer (Hank’s Buffered Saline Solution + 5% (v/v) fetal bovine serum) at 4 °C. Within 3–4 hours, bone samples were brought to the laboratory, cut into small pieces, and then digested with collagenase (type 2; 400 units/ml) for 30 minutes at 37 °C. The digest was centrifuged (600 × *g*) for 5 minutes at 4 °C, and the pellet was suspended in isolation buffer and then filtered (100-μm cell strainer) to remove bone fragments. Cells were collected from the filtrate (600 × *g*, 5 minutes), resuspended in growth media, and then seeded (5 × 10^5^ cells/cm^2^) into TCP vessels in growth media and cultured until colonies formed. Once colonies appeared, full media were removed, nonadherent cells were washed away, and fresh media were added. These cells were expanded (P1/P2) and used immediately in experiments or frozen as already described.

MSCs from 13 donors were randomly selected from our repository of 119 elderly donors (46 male, 73 female; 60–96 years old) for the current study. Only cells from 11 donors (65–86 years old) were used because MSCs from one donor did not contain enough of the four subpopulations, while cells from a second donor failed to reach confluence. In an effort to reduce variation, we only used cells from male donors. Additional studies will be necessary to confirm that the results can be generalized to both genders.

### Extracellular matrix

BM-ECM was produced under aseptic conditions using procedures developed in our laboratory [8, 10]. Briefly, BM cells were seeded (6 × 10^3^ cells/cm^2^) into six-well plates and cultured for 15 days in growth media. During the last 8 days of culture, 50 μM ascorbic acid was added to the media. The resulting ECM was washed with PBS, decellularized, washed three times with PBS followed by three additional washes with water, and then used in the experiments immediately or allowed to dry at room temperature before storage at 4 °C. If stored dry, the ECM was rehydrated immediately before use with PBS (1 hour, 37 °C).

### Colony forming unit replication assays

The MSC number and quality were determined using CFU and replication assays described previously [8, 14].

### Immunophenotyping

Mouse-anti-human nonconjugated antibodies (IgG1, IgG3, CD34, CD73, CD90, CD105, CD146, SSEA-4, Annexin V) were purchased from BD Biosciences (San Jose, CA, USA). Single cell suspensions (1 × 10^5^/100 μl) were incubated for at least 1 hour at 4 °C with primary antibody (10 μg/ml), washed twice with staining buffer (PBS + 5% FBS + 0.01% sodium azide), and then incubated with FITC conjugated goat anti-mouse IgG for 30 minutes at 4 °C. Cells were then washed twice with staining buffer and immediately analyzed (or fixed with 1% paraformaldehyde and analyzed within 72 hours) using a BD Bioscience LSRII flow cytometer running FACSDiva software. Data were analyzed and figures created using FlowJo software. At least 10,000 events/sample were measured and percent positive cells relative to isotype control were determined. The described protocol was modified slightly for assay of annexin V by treating cells with antibody suspended in dimethyl sulfoxide to permeabilize the cell membrane.

### Morphology

For assessment of cell morphology, brightfield images were taken using an Olympus IX73 Inverted Microscope (Olympus, Shinjuku, Tokyo, Japan) and analyses were performed using CellSens Dimension software (Olympus).

### Adenosine triphosphate

The ATP content of cultured cells was measured using a commercially available kit (Molecular Probes, Eugene, OR, USA) and performed as described by the manufacturer.

### Beta-galactosidase

Beta-galactosidase (β-Gal) expression was measured using a 96-well Cellular Senescence kit (Cell Biolabs, San Diego, CA, USA) and performed as described by the manufacturer.

### Cytokine arrays

Cytokine production by the cells was measured using a semiquantitative, sandwich-based, cytokine array (catalog number AAH-CYT-G5; RayBiotech, Norcross, GA, USA). Conditioned media were collected from confluent cultures after a 48-hour incubation in 1/3 volume of fresh media containing 2% FBS.

Equal volumes of conditioned media were pooled from replicate donors under each experimental condition and then assayed in duplicate per the manufacturer’s instructions. Data collection was performed by RayBiotech.

### Statistical analysis

Assays were performed in triplicate and all experiments repeated at least three times. The number of young and elderly donors for an experiment is shown in the figure legends; cells from elderly donors (*n* = 11, total number of elderly donors) were tested against cells from young donors (*n* = 5, total number of young donors), avoiding unnecessary duplication as much as possible. Data shown in the figures were pooled from independent experiments. Statistically significant differences were determined using ANOVA followed by Tukey’s test.

For flow cytometry assays, assessments were performed on one sample from each group. All experiments were repeated at least three times; the number of biological replicates is shown in the legends. Results were averaged and compared using ANOVA as already described.

For cytokine array data, duplicate assessments were performed using pooled conditioned media from three donors. Data were normalized to young MSCs on TCP per the manufacturer’s instructions. Normalized data were analyzed cytokine by cytokine using two-way repeated-measures ANOVA, followed by Tukey’s test for multiple comparisons. *P* < 0.05 was significant. Heat maps for the full array (80 cytokines) and significant senescence-associated secretory phenotype (SASP) and non-SASP cytokines were prepared using the R “gplots” package (version 3.3.3) “heatmap.2” function using *z*-score normalization for each row and hierarchical clustering based on the Euclidean distance and complete linkage method.

## Results

### Elderly and young MSCs display distinct phenotypes

**Fig. 1:**
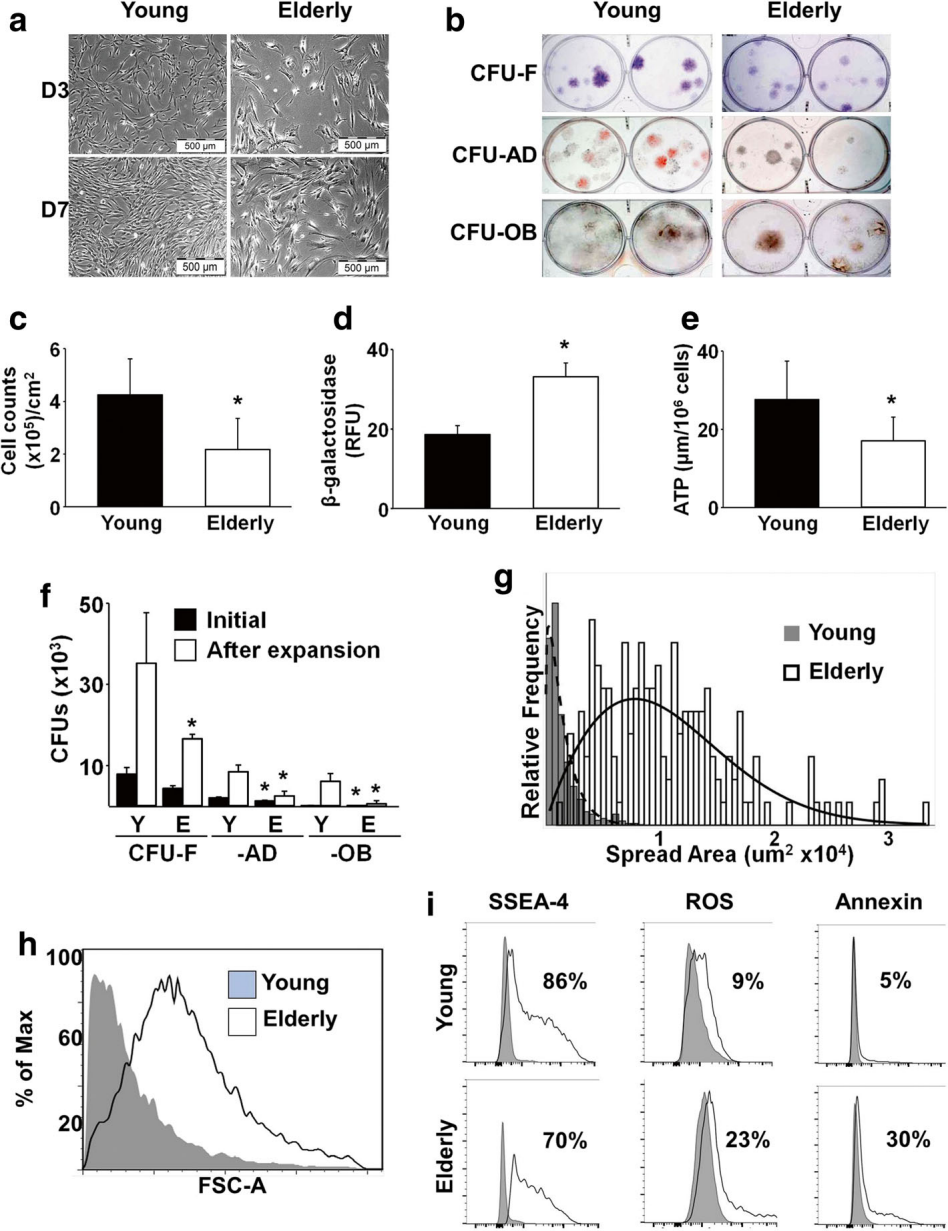
Compared to young MSCs, elderly MSC quantity and quality is reduced. **a**, **c** Brightfield microscopy of MSCs cultured on TCP for 3 or 7 days shows that elderly MSCs were less confluent than young MSCs. By day 7, density of elderly MSCs was significantly lower (*n* = 16 donors (11 elderly, five young) tested in replicate experiments). **b**, **f** After 7 days in culture on TCP, the frequency of young (Y) and elderly (E) MSCs was assessed using CFU-F, CFU-AD, and CFU-OB assays. Cells from elderly donors displayed markedly less CFU replication and differentiation capability (*n* = 10 donors (five elderly, five young) tested in replicate experiments). **d**, **e** β-galactosidase and ATP were measured and elderly MSCs were found to have significantly higher levels of β-galactosidase and significantly lower levels of ATP than young MSCs (*n* = 10 donors (five elderly, five young) tested in replicate experiments). **g**, **h** Cell spread area and cell size (using forward scatter (FSC-A) in flow cytometry) were measured after 3 days in culture. Elderly MSCs cultured on TCP were larger and displayed a wider range of mean cell spread area than young MSCs. **i** Markers of stemness (SSEA-4) and aging (intracellular ROS and Annexin V) were measured using flow cytometry. Elderly MSCs cultured for 7 days on TCP contained a smaller fraction of cells positive for SSEA-4 and a larger fraction of cells positive for early markers of apoptosis (ROS and Annexin V) than young MSCs (*n* = 10 donors (five elderly, five young) tested in replicate experiments). *P < 0.05, vs young MSCs. D day, CFU colony forming unit, F fibroblast, AD adipocyte, OB osteoblast, ATP adenosine triphosphate, ROS reactive oxygen species, SSEA-4 stage-specific embryonic antigen-4

Brightfield microscopy was used to monitor morphological differences between young and elderly MSCs during culture on TCP (Fig. 1a). Young MSCs were nearly identical in size and shape throughout the culture period, while elderly MSCs displayed large variations. After 7 days, cells were detached and plated at low seeding density for colony forming unit-fibroblast (CFU-F), CFU-adipocyte (CFU-AD), and CFU-osteoblast (CFU-OB) assays. Young MSCs formed larger and denser CFU-F colonies, as well as differentiating into greater numbers of CFU-AD and CFU-OB colonies, than elderly MSCs (Fig. 1b). By day 7, the cell density of the elderly MSCs was significantly lower than that of the young MSCs (Fig. 1c). Since increased cell size was observed in the elderly MSC cultures (Fig. 1a) and reported previously as characteristic of senescent cells [21, 24], β-galactosidase expression was measured (Fig. 1d). By day 7, elderly MSCs expressed ≈ 2-fold higher levels of β-galactosidase than young cells (*P* = 0.004), confirming that increased numbers of senescent cells were present. To further assess MSC quality, cellular ATP was measured and elderly MSCs were found to contain about half as much as young MSCs (*P* = 0.047) (Fig. 1e). MSC self-renewal was determined using a replication assay based on numbers of CFUs before and after cell expansion. Overall, CFU replication of elderly MSCs was lower than that of young cells; more importantly, CFU-AD and CFU-OB of elderly MSCs was remarkably less than that of young MSCs (Fig. 1f), suggesting that elderly MSCs had lost a significant amount of their differentiation capacity. To better quantitate the morphological differences between the two populations of MSCs, cell size and spread area were measured (Fig. 1 g, h). After 3 days in culture, elderly MSCs were > 25% more circular (less spindle-like) than young MSCs (*P* < 0.001) and had a higher mean spread area (14,326 vs 2123 μm^2^). While the mean size of the elderly MSCs appeared substantially higher than young MSCs, there was significant overlap between the two populations.

Although no differences in expression of MSC-associated surface markers were observed between the young and elderly MSC populations (Additional file 1: Figure S1), SSEA-4, originally identified on embryonic stem cells and a characteristic of actively dividing MSCs [15, 25, 26], was found to be expressed by a smaller fraction of the elderly vs young MSCs (Fig. 1i). Moreover, a larger fraction of elderly MSCs was found to express intracellular reactive oxygen species (ROS) and annexin V compared to young MSCs (Fig. 1i).

### A subpopulation of “youthful” cells can be isolated from elderly MSCs based on cell size and SSEA-4 expression

**Fig. 2:**
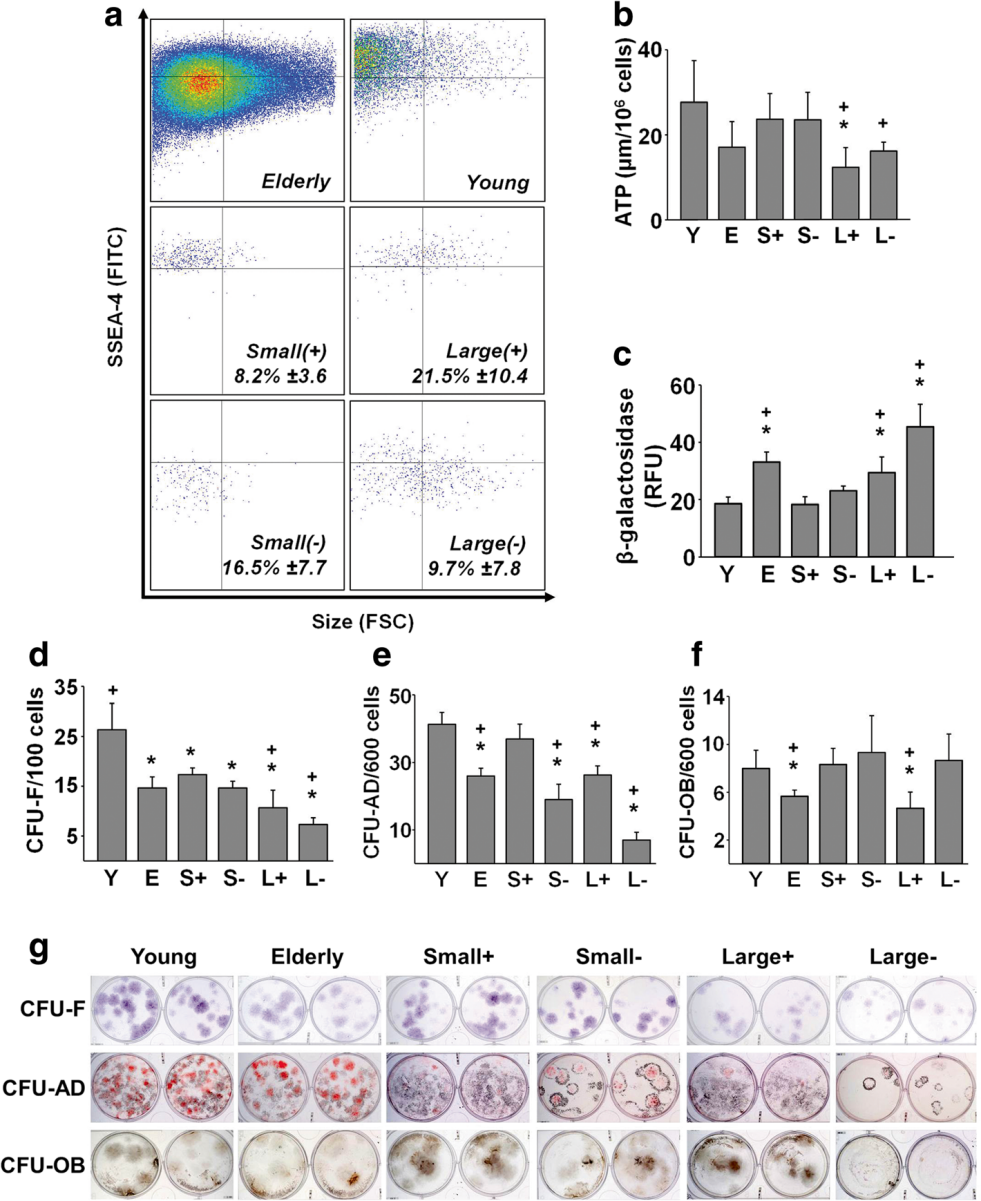
A subpopulation of elderly MSCs can be isolated using FACS that exhibits a “youthful” phenotype. **a** Flow cytometry revealed that young MSCs consisted almost exclusively of small, SSEA-4^+^ (small(+)) cells (top-right panel, upper-left quadrant), while elderly MSCs were more heterogeneous (top-left panel). After sorting elderly MSCs by size (small vs large) and SSEA-4 expression (positive vs negative), four subpopulations were obtained (lower four panels). Mean ± SD of each subpopulation shown; small(+) cells represented on average 8.2% of the elderly MSCs (*n* = 16 donors (11 elderly, five young) tested in replicate experiments). **b**, **c** After isolation, the unfractionated young and elderly MSCs and four subpopulations (S+, S–, L+, L–) were assayed for ATP content and β-galactosidase expression. Compared to elderly MSCs, ATP levels tended to be higher in young MSCs and the small(+) and small(–) subpopulations, but these differences did not achieve statistical significance. In contrast, large(+), but not large(–), cells contained significantly lower levels of ATP than young or small(+) MSCs (*P* = 0.021). β-galactosidase expression was significantly increased in the elderly MSCs and large(+) and large(–) subpopulations, compared to young and small(+) MSCs, suggesting the presence of senescent cells (*n* = 10 donors (five elderly, five young) tested in replicate experiments). **d**–**g** CFU assays (CFU-F, CFU-AD, and CFU-OB) were performed immediately after isolation to determine the enrichment of MSCs. Young MSCs consistently formed more colonies than elderly MSCs in all assays. Small(+) cells were equivalent to young MSCs in the CFU-AD and CFU-OB assays (*n* = 10 donors (five elderly, five young)). *P < 0.05, vs young MSCs; +*P* < 0.05, vs small(+) MSCs. S small, L large, Y young, E elderly, RFU relative fluorescence units, CFU colony forming unit, F fibroblast, AD adipocyte, OB osteoblast, ATP adenosine triphosphate, ROS reactive oxygen species, SSEA-4 stage-specific embryonic antigen-4

Because differences in cell size (Fig. 1g, h) and SSEA-4 expression (Fig. 1i) distinguished the young and elderly MSC populations, we explored the possibility of using these criteria to fractionate elderly MSCs into subpopulations with flow cytometry. Compared to elderly MSCs, young MSCs were a relatively homogeneous population of small SSEA-4^+^ (small(+)) cells that could be routinely identified in the upper-left quadrant in FACS (Fig. 2a). After doublet discrimination, elderly cells were segregated into four subpopulations which were then further restricted (e.g., cells with intermediate size and SSEA-4 expression were discarded) to increase the homogeneity of the populations (Fig. 2a).

The young and elderly MSCs and the four subpopulations of elderly MSCs were evaluated for their ATP content and β-galactosidase expression (Fig. 2b, c). Small(+) and small(–) cells contained levels of ATP equivalent to that of young MSCs. In contrast, large(+) and large(–) MSCs were found to have ATP levels equivalent to that of the original elderly MSCs and roughly half that of young and small(+) MSCs. These results suggest that the quality of the small(+) and small(–) cells is superior to that of the parent elderly MSCs and both subpopulations of large MSCs.

To further determine which of the subpopulations of elderly cells were enriched in MSCs, we seeded the same number of cells onto TCP for CFU assay (Fig. 2d–f). Both small(+) and small(–) cells contained more CFU-F than either of the large cell subpopulations. The number of CFU-AD and CFU-OB generated by small(+) cells was very similar to that generated by young MSCs and significantly greater than that generated by both the elderly MSCs and large(+) cells (Fig. 2d–f). The visual appearance of the colonies formed in the three CFU assays are shown in Fig. 2 g.

### Elderly MSCs secrete factors responsible for inhibiting young MSC proliferation

**Fig. 3:**
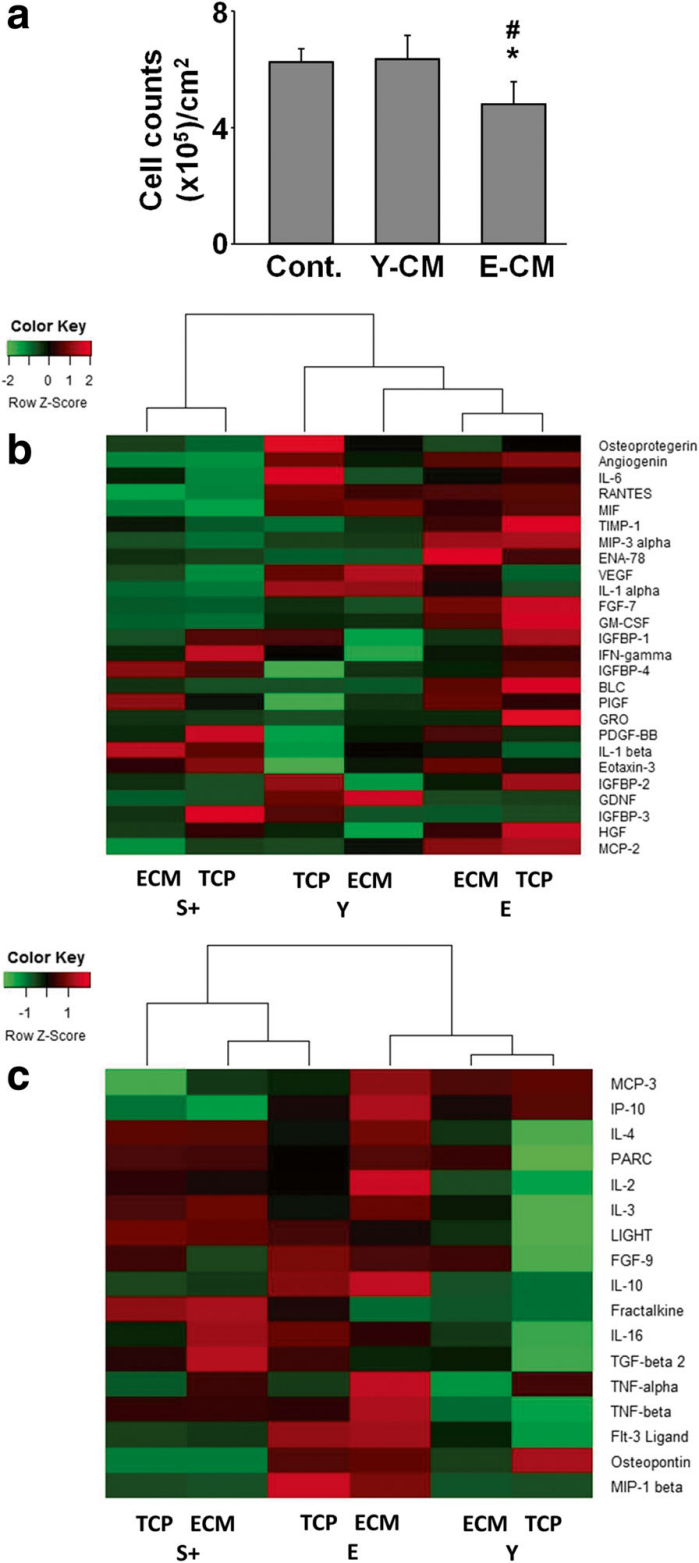
Elderly MSCs produce cytokines, associated with SASP, capable of inhibiting young MSC proliferation. **a** Conditioned media (CM) were collected from 7-day cultures of young and elderly MSCs on TCP and then added to naïve cultures of young MSCs at a ratio of 1 part CM:2 parts fresh media. Proliferation after 7 days was less in cultures treated with CM from elderly MSCs, suggesting the presence of inhibitory factors (*n* = 6 donors (three elderly, three young)). **b** CM from young, small(+), and elderly MSCs cultured for 7 days on TCP or ECM were analyzed using a cytokine array and a heatmap of the SASP-associated cytokines prepared. Small(+) cells expressed less SASP-related cytokines than either young or elderly MSCs. Assays were performed in duplicate using pooled CM (*n* = 3 donors/group). **c** CM were collected, analyzed as in (**b**), and a heatmap of the non-SASP-associated cytokines prepared. Non-SASP-related cytokine production by small(+) cells was similar to that of elderly MSCs, suggesting that some characteristics of the elderly heritage remain. Assays were performed in duplicate using pooled CM (*n* = 3 donors/group). *P < 0.05, vs CM from young MSCs; #*P* < 0.05, vs control media. S small, Y young, E elderly, ECM extracellular matrix, TCP tissue culture plastic

Next, conditioned media were collected to determine whether factors secreted by elderly MSCs were able to affect young MSCs. Media collected from elderly MSC cultures were found to significantly inhibit proliferation of young MSCs vs control media or media of young MSCs (*P* = 0.004 or *P* = 0.007, respectively) (Fig. 3a).

To identify specific factors responsible for inhibiting the young MSCs, conditioned media from elderly, young, and small(+) MSCs were analyzed using a commercially available cytokine array (catalog number AAH-CYT-G5; RayBiotech). The array is capable of detecting a total of 80 cytokines (Additional file 2: Figure S2). Hierarchical cluster analysis showed that small(+) MSCs, cultured on TCP or ECM, were distinct from the parent elderly MSC population but similar to the young MSCs (Additional file 3: Figure S3). In addition, the array tests for 44 cytokines previously identified as being characteristic of the SASP [27], and conditioned media from elderly MSCs contains 22 of these SASP cytokines (19 were significantly increased and three significantly decreased compared to young MSCs) (Fig. 3b). However, in the conditioned media of small(+) cells, SASP-related cytokine production was reduced vs elderly MSCs and similar to that of young MSCs (no significant difference between young and small(+) MSCs, *P* = 0.068), further suggesting that the small(+) cells have “youthful” phenotypic characteristics. It is noteworthy that young MSCs produced less IL-6 (an inflammation-related cytokine) when cultured on ECM vs TCP. In addition, young MSCs produced more vascular endothelial growth factor (VEGF) and glial cell-derived neurotrophic factor (GDNF) when maintained on ECM vs TCP. The former is able to stimulate angiogenesis, and the latter is involved in preventing motor neuron apoptosis. Interestingly, the profile of non-SASP-associated cytokines released by the small(+) cells was relatively similar to that of the parent elderly MSCs (Fig. 3c), suggesting that the elderly background (“heritage”) of the small(+) cells was still being expressed.

### Small SSEA-4^+^ cells retain their “youthful” phenotype after expansion on young ECM

Since small(+) cells displayed a “youthful” phenotype, the next challenge was to develop a strategy for expanding these cells while maintaining their phenotype. To accomplish this, we used a 3D native ECM, generated by bone marrow stromal cells from young donors (~20 years old), which promotes MSC expansion and preserves “stemness” and differentiation capacity [15].

**Fig. 4:**
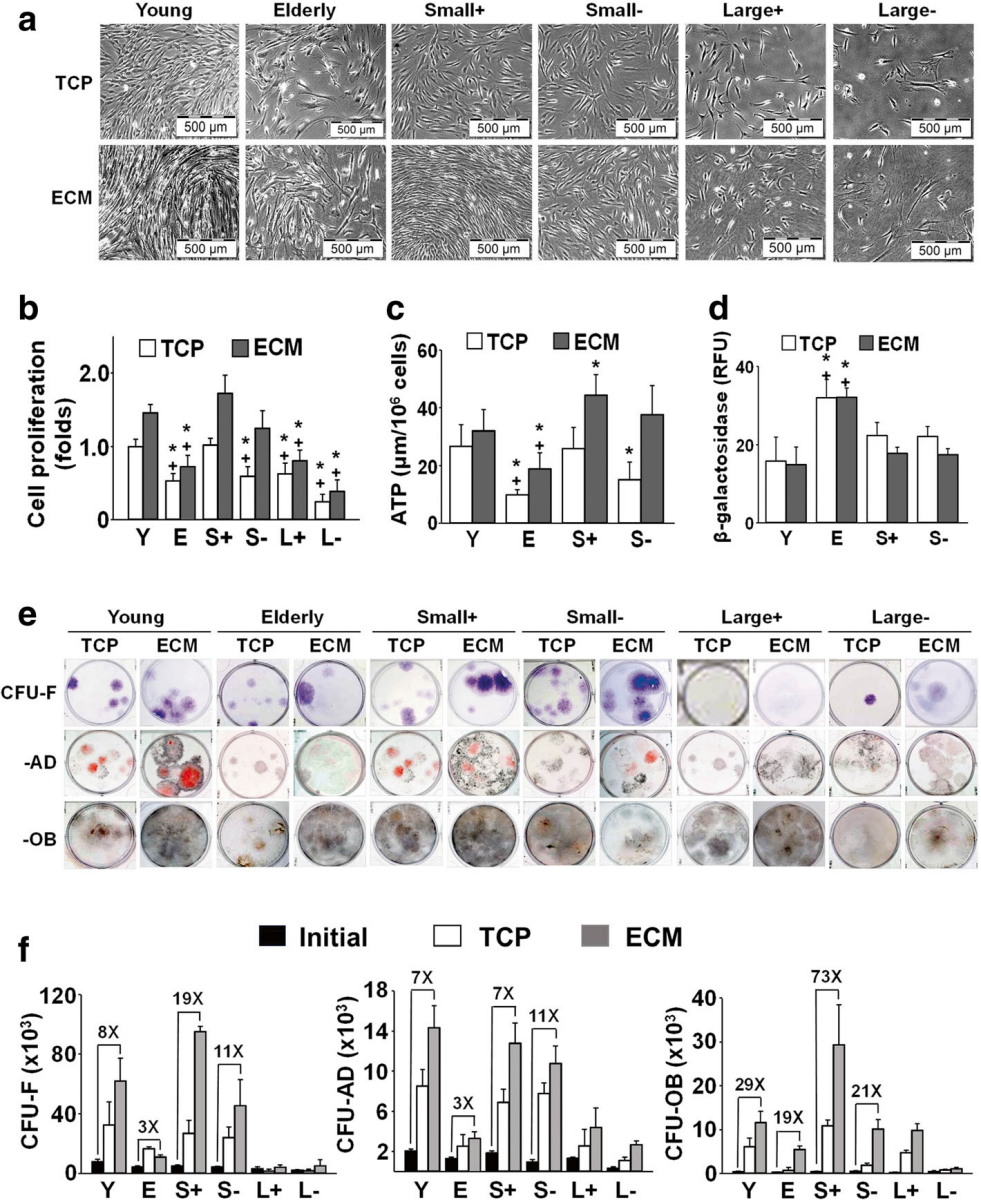
Expansion of elderly MSC subpopulations on ECM increases cell number and preserves stemness of small(+) cells. **a**, **b** Brightfield microscopy of young and elderly MSCs and isolated subpopulations cultured for 7 days on TCP or ECM revealed that growth on ECM significantly enhanced proliferation of young MSCs and both types of small MSCs. Proliferation was calculated as a fold-change by normalizing cell counts at the end of culture to young cells on TCP (*n* = 16 donors (11 elderly, five young) tested in replicate experiments). **c**, **d** ATP levels, but not β-galactosidase expression, were significantly increased in small(+) and small(–) cells and elderly MSCs with culture on ECM (vs TCP) for 7 days, suggesting that ECM promoted maintenance of cell metabolism and inhibited senescence. There were insufficient numbers of large(+)/large(–) cells for assay (*n* = 10 donors (five elderly, five young) tested in replicate experiments). **e**, **f** Following culture on TCP or ECM for 7 days, young and elderly MSCs and isolated subpopulations were detached and seeded at clonal density on TCP for CFU replication assays (CFU-F, CFU-AD, and CFU-OB). The CFU results were consistent with the proliferation data. Culture of young MSCs on ECM significantly enhanced CFU-AD and CFU-OB production, but not CFU-F. Small(+) cells similarly displayed a significant increase in CFU-AD and CFU-OB, as well as CFU-F, production with culture on ECM. CFU replication was calculated by determining the number of CFUs post culture on ECM or TCP and dividing by the number of CFUs produced by the initial population of cells. Fold increases over the initial number of CFUs are shown (*n* = 10 donors (five elderly, five young) tested in replicate experiments). *P < 0.05, vs young MSCs; +*P* < 0.05, vs small(+) MSCs. S small, L large, Y young, E elderly, ECM extracellular matrix, TCP tissue culture plastic, ATP adenosine triphosphate, RFU relative fluorescence units, CFU colony forming unit, F fibroblast, AD adipocyte, OB osteoblast

Young and elderly MSCs and fractionated elderly MSCs were plated at the same seeding density on TCP or ECM and cultured for 7 days (Fig. 4a). Morphologically, young MSCs and small(+) and small(–) MSCs maintained on both culture surfaces had a uniform size and shape; in addition, there was a consistently greater number of cells found with culture on ECM vs TCP. In contrast, elderly, large(+), and large(–) MSCs grew more slowly.

Cell proliferation revealed a similar growth pattern between young MSCs and the small(+) cells expanded on either TCP or ECM (Fig. 4b), although proliferation of young MSCs and small(+) and small(–) cells on ECM was higher than on TCP (*P* < 0.05). In contrast, proliferation of elderly MSCs and the large(+) and large(–) cells was significantly lower than the young MSCs and no differences were noted between culture on ECM vs TCP.

To determine the quality of the two small subpopulations, cellular ATP and β-galactosidase expression were measured (Fig. 4c, d). Compared to young MSCs, elderly MSCs had significantly lower ATP levels and greater expression of β-galactosidase after culture on either TCP or ECM. In contrast, small(+) cells had the same level of ATP and β-galactosidase as the young MSCs after culture on TCP; however, small(+) cells had significantly higher ATP levels and decreased β-galactosidase expression after culture on ECM. Interestingly, the small(–) cells behaved like the small(+) cells, although differences varied in terms of statistical significance.

For CFU replication assays, cells were detached after 7 days in culture and reseeded at clonal density for determination of CFU-F, CFU-AD, and CFU-OB (Fig. 4e, f). Small(+) and small(–) cells post-expanded on ECM formed colonies that were larger, denser, and more numerous than on TCP and displayed increased adipogenic and osteogenic potential (Fig. 4e). This was not the case for the large cell populations which formed fewer CFUs, although large(+) cells displayed increased osteogenic potential after culture on young ECM (Fig. 4e). In Fig. 4f, results of the CFU replication assays showed that small(+) cells maintained on ECM increased by 19-fold, 7-fold, and 73-fold in CFU-F, CFU-AD and CFU-OB, respectively. In contrast, elderly cells maintained on ECM only showed an increase of 3-fold, 3-fold, and 19-fold. Replication of CFUs by both of the large cell subpopulations was lower than that of the two small subpopulations of elderly cells, and was even lower than the unfractionated elderly MSCs.

### Small SSEA-4^+^ cells retain their “youthful” phenotype through multiple passages in culture

**Fig. 5:**
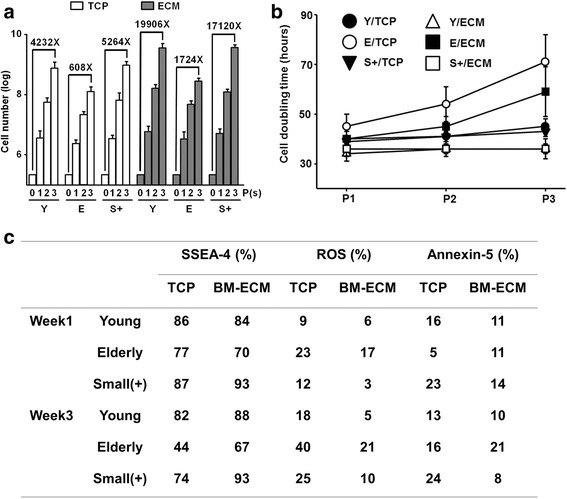
Small(+) cells retain their “youthful” phenotype through multiple passages on ECM. Young (Y), elderly (E), and small(+) (S+) MSCs were seeded at 2000 cells/cm^2^ onto TCP or ECM and subcultured in growth media for 3 weeks. Cells were passaged every 7 days and reseeded at the same density used initially (*n* = 10 donors (five elderly, five young) tested in replicate experiments). **a** Total number of cells obtained after culture increased with each passage and was dependent on culture surface and cell type. Compared to culture on TCP, culture on ECM for three passages dramatically increased the number of Y and S+ cells (19,906-fold and 17,120-fold, respectively). As expected, the yield of E cells was much lower. **b** Cell doubling time was affected by cell type, culture surface, and passage number. At P3, Y and S+ cells had shorter doubling times on TCP than E cells and culture on ECM retained shorter doubling times (S+ < Y < E). **c** Immunophenotypic properties of Y and S+ cells were maintained through P3. Both Y and S+ cells maintained high expression of SSEA-4 and low levels of ROS and annexin V with culture on ECM. BM-ECM bone marrow-derived extracellular matrix, TCP tissue culture plastic, P passage, ROS reactive oxygen species, SSEA-4 stage-specific embryonic antigen-4

To establish a stem cell bank of high-quality autologous MSCs for the elderly, we determined whether culturing the small(+) cells through three passages (P3) would have a deleterious effect on their stem cell properties (Fig. 5). Young, elderly, and small(+) MSCs were seeded at 2000 cells/cm^2^ onto TCP or ECM and subcultured for 3 weeks (1 week/passage). Each week cells were harvested, counted, and an aliquot reseeded using the same seeding density. The accumulated total number of cells at each passage is shown in Fig. 5a. The results showed that young and small(+) MSCs proliferated to similar extents on both TCP and ECM, but the fold increase was consistently greater on the ECM. For small(+) cells, there was a 17,120-fold increase (over the initial number of cells seeded) on ECM vs a 5264-fold increase on TCP over P3. In contrast, the number of elderly MSCs increased 1724-fold on ECM compared to 608-fold on TCP. As expected, the total number of cells accumulated over time was related to the doubling time of the cultures (Fig. 5b). Both young and small(+) MSCs cultured only on ECM maintained stable and equivalent doubling times through P3 which were shorter than observed on TCP (34–36 vs 39–44 hours, respectively). In contrast, doubling times for the elderly MSCs cultured on TCP or ECM were substantially longer at 45–70.9 hours and 39.9–59.1 hours, respectively (Fig. 5b). To determine whether stem cell quality was preserved during passage, the cells were subjected to immunophenotypic analyses at P1 and P3 (Fig. 5c). As observed with young MSCs cultured on ECM, small(+) cells maintained high expression of SSEA-4 through P3 and relatively low levels of ROS and annexin-5, compared to maintenance on TCP, and elderly cells maintained on ECM or TCP.

## Discussion

At present, criteria for identifying and characterizing MSCs follow those proposed by the ISCT [28]. Accordingly, MSCs are defined as cells that adhere to TCP under standard culture conditions, express specific cell surface antigens (> 95% positive for CD73, CD90, and CD105), and have trilineage differentiation potential. As reported by others [25, 26, 29], and shown in the present study, these criteria are inadequate. First, plastic adhesion of very early-stage MSCs is poor [29, 30] and accumulating evidence indicates that culture on TCP alters the MSC phenotype [10, 31, 32]. Second, cell surface marker expression, which is routinely used to define MSCs, does not correlate well with the differentiation state of the cells or their potency, and does not distinguish between MSCs obtained from young versus old donors (Additional file 1: Figure S1). These shortcomings are highlighted in the current study.

In the present study, we have uncovered several characteristics that correlate well with functional properties of the cells. With aging, loss of stemness was accompanied by a decrease in cell proliferation and differentiation, SSEA-4 expression, and ATP content, and an increase in cell size and expression of β-Gal, intracellular ROS, and Annexin-V. However, unlike our previous report in mice [15], the present study showed that exposure to young ECM only modestly restored the proliferation and differentiation of elderly MSCs (Fig. 4b). Interestingly, the percentage of SSEA-4 in elderly MSCs cultured on young ECM was rescued, but without increasing the total number of cells, suggesting that cell death is occurring at a higher rate in the elderly cells. To confirm that elderly MSCs produce cytokines with deleterious paracrine effects, we treated young MSCs with media conditioned by elderly cells and found that proliferation was suppressed (Fig. 3a). Subsequently, we found that conditioned media from elderly MSC cultures contained significantly increased levels of cytokines associated with the SASP [27]. We hypothesize that, in vivo, small(+) cells fail to thrive because they are exposed to various SASP cytokines which negatively impact their behavior [23, 33–35].

Prior work on the SASP has been performed on fetal cells or immortalized cell lines [27, 36–38]. To the best of our knowledge, the current study is the first to report that elderly MSCs display the SASP. This observation may have great relevance for stem cell-based regenerative therapies, since the prevailing logic is that MSCs have potent immunomodulatory capabilities and should be able to combat inflammation and reverse the effects of the SASP in the elderly population [39, 40]. In contrast, we show that elderly MSCs, which contain a considerable number of senescent cells, may actually contribute to inflammation and further diminish endogenous MSC function. Thus, autologous stem cell therapies in elderly individuals may actually compound or promote age-related degeneration. In the present study, we show that in contrast to the original population of elderly MSCs, the “youthful” subpopulation expresses fewer SASP cytokines at levels similar to young MSCs (Fig. 3b), but also displays a non-SASP cytokine profile reminiscent of the parent elderly population (Fig. 3c). These findings support our hypothesis that “youthful” MSCs can be rescued from the deleterious environment in the elderly MSC population. Interestingly, the cytokine array also called to our attention that conditioned media of young MSCs contained less IL-6 and more VEGF and GDNF when cultured on ECM vs TCP, suggesting that the therapeutic efficacy of MSCs may be enhanced by maintenance on ECM.

While the results are promising, one drawback of the study is that cells from young and elderly donors were obtained from different sources. Young MSCs were obtained from iliac crest BM aspirates, while elderly MSCs were obtained from surgical waste (bone chips, BM, etc.) of patients undergoing total joint arthroplasty. Thus, there were substantial differences in age, donor site (femur vs iliac crest), and presence/absence of disease between the groups. These issues do not significantly detract from our findings as we were able to obtain MSCs with characteristics consistent with other reports in the literature. However, it will be necessary in the future to address these potential issues in “healthy” elderly donors using an appropriate animal model.

**Fig. 6:**
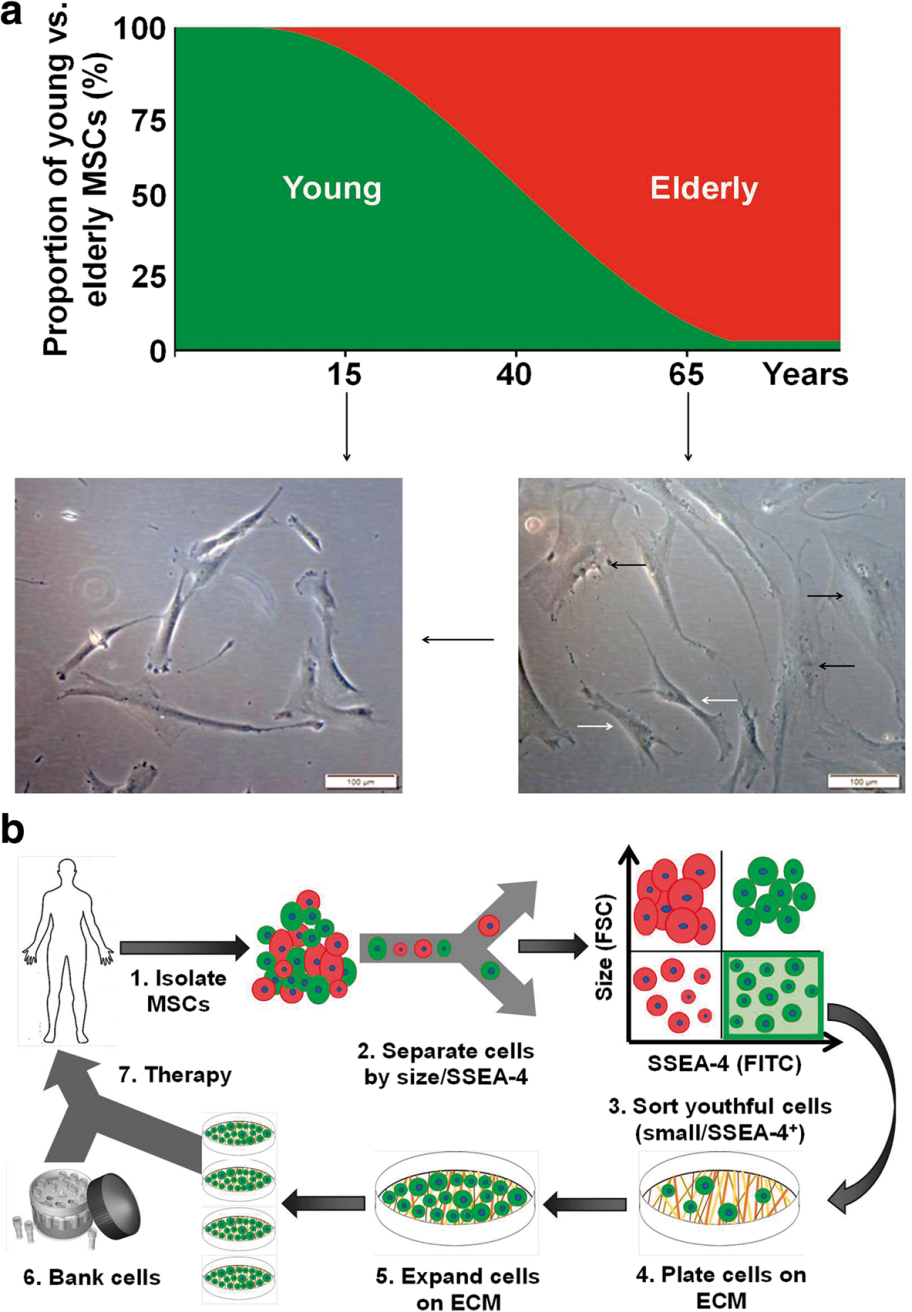
Elderly MSCs with a “youthful” phenotype can be expanded on young ECM for cell-based therapies. **a** Ratio of young to elderly MSCs changes with chronological age. In the elderly, the proportion of young (“youthful”) MSCs has dropped to approximately 8–10% of the total population. This can be clearly seen in the brightfield micrographs. In the young, virtually all cells display a fibroblastic spindle-shaped morphology. With advancing age, cells become larger and spread across the surface (black arrows), but fibroblastic cells can still be found (white arrows). **b** Our current studies strongly suggest it is possible to rescue these “youthful” cells by separation from the parent MSC population, based on cell size and SSEA-4 expression, and then expand them on a young ECM to bank large quantities of high-quality autologous MSCs for more effective treatment of age-related diseases. ECM extracellular matrix, FSC forward scatter, MSC mesenchymal stem cell, SSEA-4 stage-specific embryonic antigen-4

Recently, others have confirmed that MSCs maintained on native ECM (vs TCP) are smaller in size and of higher quality [16]. In the current study, we were able to consistently isolate a subpopulation of “youthful” cells from preparations of elderly MSCs using these criteria and then successfully expand them on a previously characterized culture system that promotes proliferation and retention of stemness [41]. The number of cells obtained at the end of 3 weeks was more than 28-fold higher than when unfractionated elderly MSCs were cultured on TCP (fold-changes: 17,120/608) (Fig. 5a). In Fig. 6, we propose that the ratio of young to elderly MSCs changes with aging and outline a strategy for establishing personal stem cell banks with sufficient numbers of high-quality cells to support multiple infusions of autologous “youthful” MSCs. Based on data in the current study, 2.5 × 10^6^ BM-MSCs from an 80-year-old 90-kg donor would be expected to contain about 2 × 10^5^ small(+) cells. After 3 weeks of subculture on BM-ECM (initial seeding density of 2 × 10^3^ cells/cm^2^), we would expect that the small(+) cell population would have increased to approximately 3 × 10^9^ cells. To treat this patient (donor) with an infusion of 1 × 10^6^ cells/kg every 3 months, 3 × 10^9^ cells would support more than 32 infusions of cell-based therapy over 8 years. To the best of our knowledge, there is no currently available system able to rapidly amplify such a small number of cells from an elderly patient and meet the clinical demand for large numbers of high-quality MSCs. This approach would be especially valuable for patients requiring multiple infusions to treat age-related degenerative diseases that are virtually unaffected by a single infusion of stem cells [12]. Furthermore, repeated infusion of high-quality MSCs would provide a strategy for slowing down or reversing the well-known deleterious effects of aging on the microenvironment and its effects on stem cell viability and function/activity. However, a significant amount of work remains to achieve this goal, including the optimization of cell dose, length of treatment, and route of administration (systemic vs local), and enable the gradual reversal of the aged MSC microenvironment.

## Conclusions

The results reported here suggest that it is feasible to establish personal stem cell banks for the elderly containing large numbers of high-quality autologous MSCs. The availability of sufficient numbers of high-quality autologous cells has the potential to dramatically improve clinical outcome(s) by making multiple administrations/infusions of cells possible and overcoming the age-related decline in stem cell quality and attenuate the effects of aging on the stem cell niche.

## Additional files

Additional file 1: Figure S1. Showing MSC surface marker expression was independent of donor age and culture substrate. Expression of CD73, CD90, and CD105 was greater than 95% in each group. (TIF 373 kb)

Additional file 2: Figure S2. Showing cytokines assayed in the conditioned media of young, elderly, and small(+) MSCs cultured on TCP or ECM substrates. (TIF 4048 kb)

Additional file 3: Figure S3. Showing heat map representation of cytokine release by young, elderly, and small(+) MSCs cultured on TCP or ECM substrates. (TIF 3807 kb)

## Abbreviations

BM-ECM: Bone marrow-derived extracellular matrix
BM-MSC: Bone marrow-derived mesenchymal stem cell
GDNF: Glial cell-derived neurotrophic factor
PBS: Phosphate buffered saline
ROS: Reactive oxygen species
SASP: Senescence-associated secretory phenotype
SSEA-4: Stage-specific embryonic antigen-4
TCP: Tissue culture plastic/polystyrene
VEGF: Vascular endothelial growth factor
