# Supplementary figures and images for: Restoring the quantity and quality of elderly human mesenchymal stem cells for autologous cell-based therapies
Source: Stem Cell Res Ther. 2017 Oct 27;8:239. doi: 10.1186/s13287-017-0688-x (PMC5658952; doi:10.1186/s13287-017-0688-x)

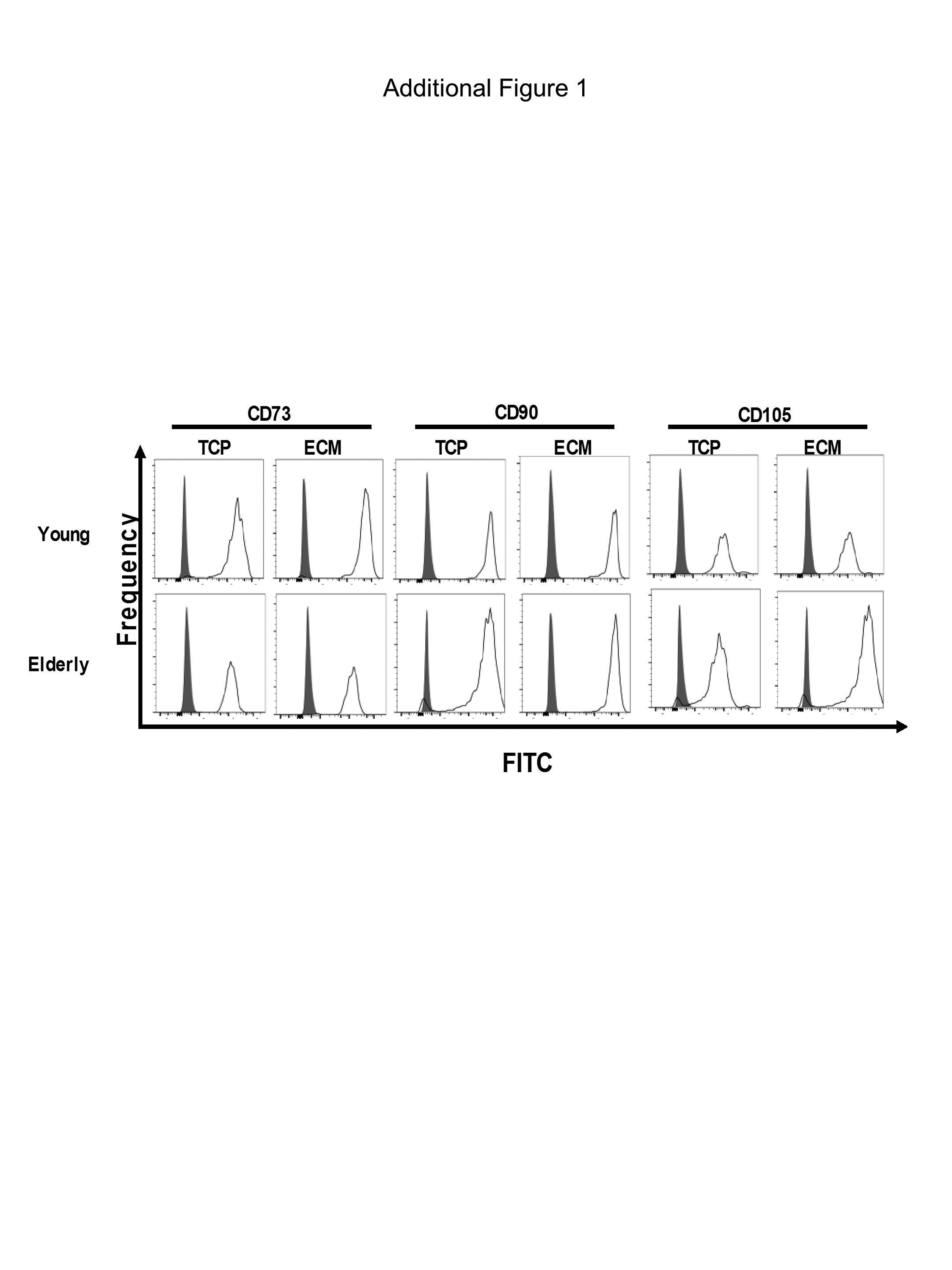

Supplement: Supplementary file 1 — Showing MSC surface marker expression was independent of donor age and culture substrate. Expression of CD73, CD90, and CD105 was greater than 95% in each group. (TIF 373 kb) [file 13287_2017_688_MOESM1_ESM.tif]

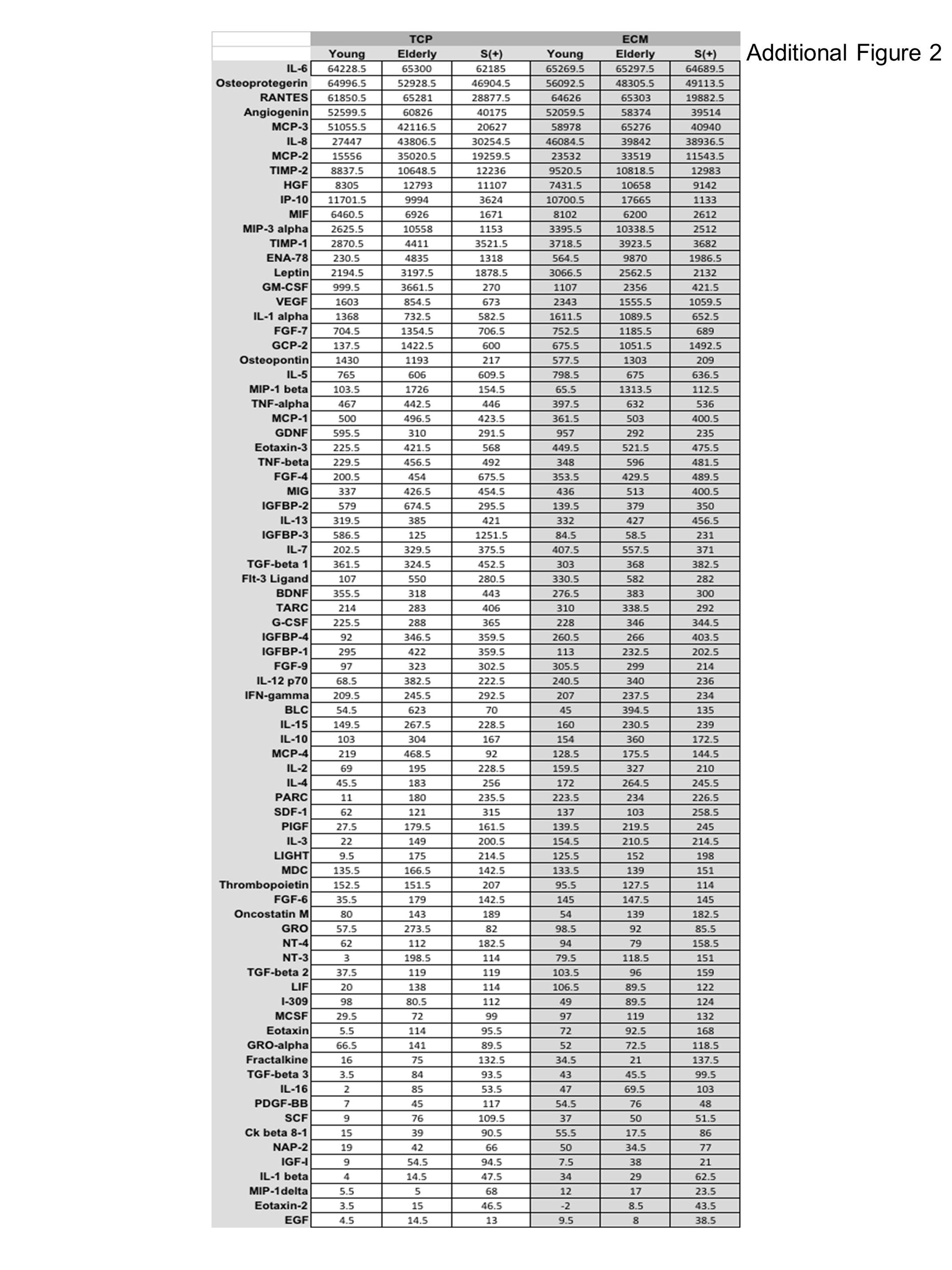

Supplement: Supplementary file 2 — Showing cytokines assayed in the conditioned media of young, elderly, and small(+) MSCs cultured on TCP or ECM substrates. (TIF 4048 kb) [file 13287_2017_688_MOESM2_ESM.tif]

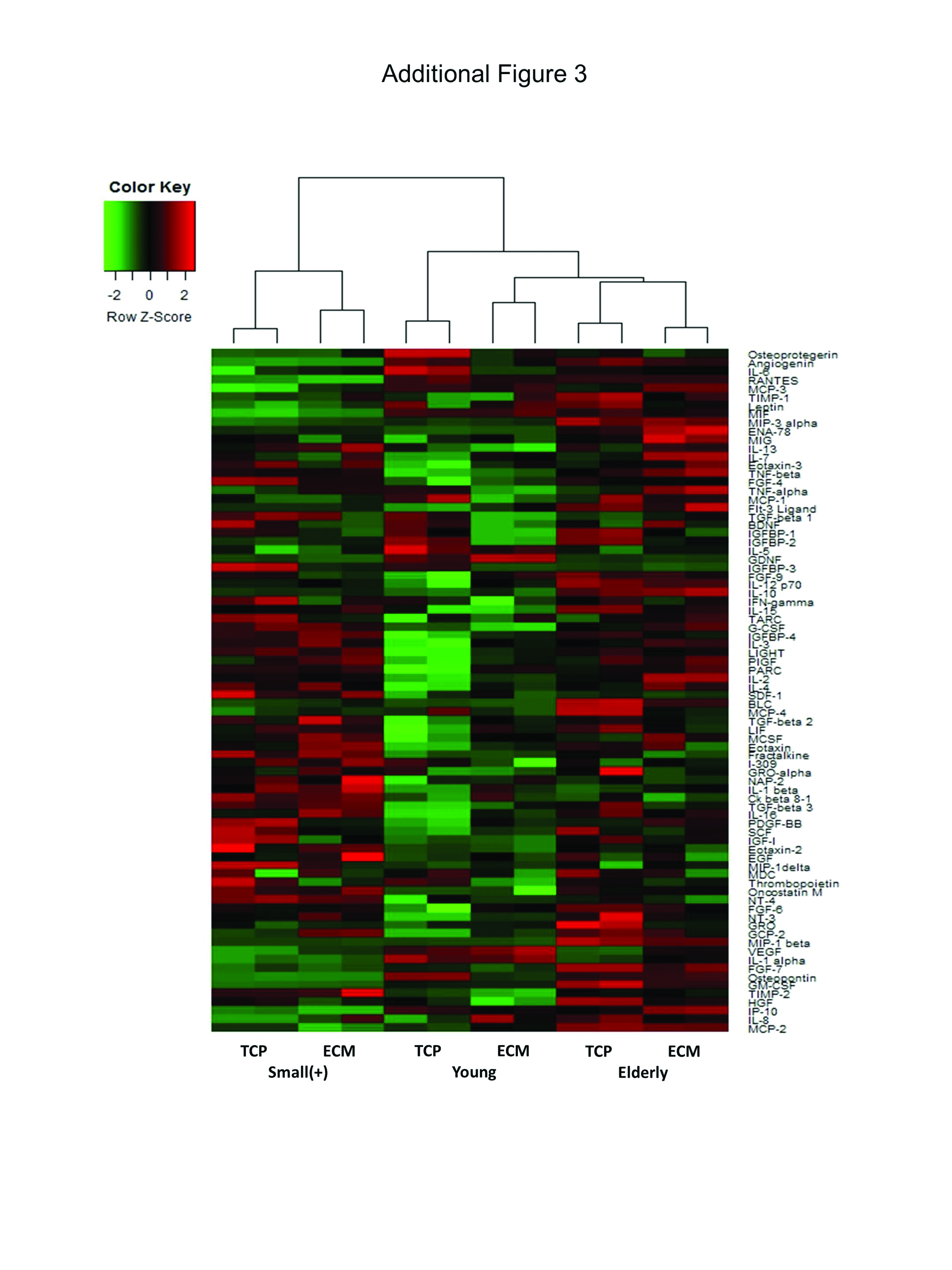

Supplement: Supplementary file 3 — Showing heat map representation of cytokine release by young, elderly, and small(+) MSCs cultured on TCP or ECM substrates. (TIF 3807 kb) [file 13287_2017_688_MOESM3_ESM.tif]
